# Optical demonstration of quantum fault-tolerant threshold

**DOI:** 10.1038/s41377-022-00891-9

**Published:** 2022-07-05

**Authors:** Kai Sun, Ze-Yan Hao, Yan Wang, Jia-Kun Li, Xiao-Ye Xu, Jin-Shi Xu, Yong-Jian Han, Chuan-Feng Li, Guang-Can Guo

**Affiliations:** 1 CAS Key Laboratory of Quantum Information, University of Science and Technology of China, Hefei, 230026 China; 2 CAS Center for Excellence in Quantum Information and Quantum Physics, University of Science and Technology of China, Hefei, 230026 China; 3 Hefei National Laboratory, University of Science and Technology of China, Hefei, 230088 China

**Keywords:** Quantum optics, Nonlinear optics

## Abstract

A major challenge in practical quantum computation is the ineludible errors caused by the interaction of quantum systems with their environment. Fault-tolerant schemes, in which logical qubits are encoded by several physical qubits, enable to the output of a higher probability of correct logical qubits under the presence of errors. However, strict requirements to encode qubits and operators render the implementation of a full fault-tolerant computation challenging even for the achievable noisy intermediate-scale quantum technology. Especially the threshold for fault-tolerant computation still lacks experimental verification. Here, based on an all-optical setup, we experimentally demonstrate the existence of the threshold for the fault-tolerant protocol. Four physical qubits are represented as the spatial modes of two entangled photons, which are used to encode two logical qubits. The experimental results clearly show that when the error rate is below the threshold, the probability of correct output in the circuit, formed with fault-tolerant gates, is higher than that in the corresponding non-encoded circuit. In contrast, when the error rate is above the threshold, no advantage is observed in the fault-tolerant implementation. The developed high-accuracy optical system may provide a reliable platform to investigate error propagation in more complex circuits with fault-tolerant gates.

## Abstract (web summary)

We observe the error rate threshold of quantum fault-tolerant computation by using optical spatial modes of two entangled photons to encode logical qubits.

## Graphical abstract

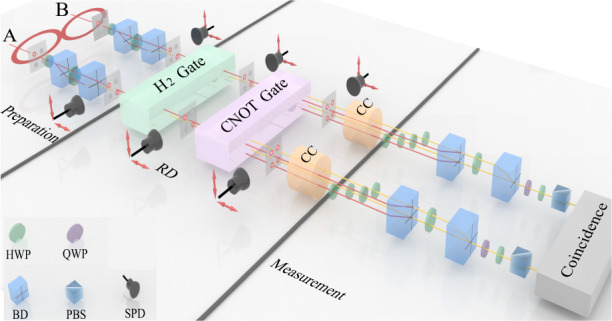

## Introduction

Error is inevitable in practical quantum computing during encoding, operation, and decoding processes. Although quantum error has been experimentally investigated^1–12^ in different physical systems and high-fidelity quantum gates are achieved^13–15^, the experimental demonstration of a complete fault-tolerant computation^16–20^ remains still a great challenge^21^. In a full fault-tolerant implementation, quantum information processing, particularly including a set of universal quantum computation gates, is additionally protected against errors. The correct probability of the output from the encoded fault-tolerant quantum circuit is higher than that from the corresponding non-encoded circuit if the error rate of the underlying hardware is below a threshold^22–27^. Therefore, fault-tolerant encoding may be essential to achieve future large-scale quantum computation^28,29^.

Practical quantum advantage may be achievable even under the presence of errors by applying the noisy intermediate-scale quantum (NISQ) technology^30^. Although a complete fault-tolerant computation with practical application is still beyond the reach of NISQ technology, fault-tolerant quantum circuits could be demonstrated in a small system^21,31,32^ to show the effectiveness of noise mitigation in logical qubits and even implement some practical applications with NISQ technology^33^.

In ref. ^21^ a special fault-tolerant protocol is proposed for a small system consisting of five qubits, of which one is regarded as an ancillary qubit and the others are used to encode logical qubits. In this protocol, encoding, decoding, and some gates, such as single-qubit Pauli operators *σ*^*x*^ and *σ*^*Z*^, and the two-qubit controlled-not (CNOT) operator (these Clifford operators are not universal), can be implemented fault tolerantly in logical space with the help of post-selection. It is shown that errors in this protocol cannot be corrected. Following this protocol, fault-tolerant error detection of encoding has been demonstrated in trapped ions^34^, superconducting qubits^35^, and the IBM quantum devices^36–38^. However, the key aspect of the quantum circuit implementing with fault-tolerant operations, that is, the existence of the threshold of error rate below which the circuit is realized in a fault-tolerant manner, has not been explicitly demonstrated.

In this work, we experimentally demonstrate the threshold of error rate for quantum circuits formed with fault-tolerant gates implemented in an all-optical setup. Based on the encoding method, we encode two logical qubits using four qubits which are mapped to the optical path information of two entangled photons. Besides the preparation stage, we experimentally implement a single-qubit Hadamard gate and a two-qubit CNOT gate in the logical space to form a complete quantum circuit in which error gates are imported based on the bit-flip error. When comparing the output probabilities of the encoded circuit and those of non-encoded circuit, we could determine the fault-tolerant threshold of the error rate. Our results clearly demonstrate that when the error rate remains below the threshold, the probability to obtain correct output results in the fault-tolerant circuit is higher than that of the corresponding non-encoded circuit. On the other side, if the error rate is above the threshold, no benefit is obtained from the fault-tolerant implementation.

## Results

**Fig. 1:**
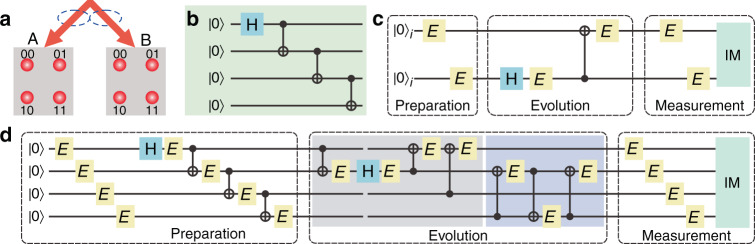
Quantum circuits with fault-tolerant gates under the presence of errors. **a** Spatial modes on each side, A and B which are bipartite entangled, are marked as |00〉, |01〉, |10〉, and |11〉 (ket symbols are omitted for brevity). The basis of four physical qubits, say |0001〉, is denoted as the coincidence count between the mode |00〉 on A side and the mode |01〉 on the B side. **b** The circuit of preparing logical qubit state |00〉_*l*_ from the initial state |0000〉. **c** Non-encoded circuit includes stages of the preparation, evolution, and measurement starting from logical state |00〉_*l*_ with errors. Hadamard gate (H) is applied to the second logical qubit followed by a CNOT gate CNOT_21_. Error gates *E* are imported throughout the circuit. IM denotes ideal measurement. **d** The complete fault-tolerant circuit implementing logical operations H_2_ and CNOT_21_. In CNOT operation, error gate *E* affects only the target qubit

Following the encoding protocol introduced in the Materials and Methods, two logical qubits are encoded with four physical qubits, where the encoded space only involves an even number of |1〉 in physical qubits. The four physical qubits are mapped to coincident modes of two entangled photons. This method of mapping the qubits to optical spatial modes could simulate the operation of the individual qubit with the evolution of spatial modes^39^. By coherently adjusting spatial modes, single- and two-qubit gates can be conveniently realized. Logical state $\left| {00} \right\rangle _l \, = (\left| {0000} \right\rangle + \left| {1111} \right\rangle )/\sqrt 2$ can be fault tolerantly prepared with post-selection following the circuit presented in Fig. 1b starting from the initial physical state |0000〉. In this protocol, a set of quantum gates, such as *σ*^*x*^, Hadamard, and CNOT gates, operated on logical qubits can be implemented in a fault-tolerant manner. As a result, a circuit, only formed by these fault-tolerant gates, is implemented fault tolerantly and there exists a threshold of the error rate. Our main task is to experimentally demonstrate the existence of the threshold in the fault-tolerant circuit.

A complete fault-tolerant circuit includes preparation, evolution (containing a set of gates), and measurement, and an error may occur at any stage of the circuit. For simplicity, in the Materials and Methods, we describe an operator with an error on qubits by its ideal quantum operation followed by an error gate E (assumed to be the same for all the operations). The noisy measurement can be decomposed into error operation E and the ideal measurement (IM). As illustrated in Fig. 1c (non-encoded circuit), the evolution stage consists of a Hadamard operation on the second logical qubit, H_2_, followed by a two-qubit CNOT operation CNOT_21_ in which the first (target) qubit is controlled by the second qubit. Figure 1d shows the corresponding encoded circuit implementing these two logical operations.

Errors in preparation, evolution, and measurement, are considered in both non-encoded and encoded circuits, in which the error gate is assumed to be *E* = *σ*^*x*^ with error rate ϵ = 1 − *p* and *p* being the correct probability, thus establishing a coherent error. Experimentally controlling and identifying the error rate ϵ in each gate is the key process in our study. The probability of successful output can be defined as *F*_*p*_ = Tr[*ρ*_ideal_. *ρ*_exp_] (a function of *p*), where *ρ*_ideal_ and *ρ*_exp_ are ideal and experimental output states, respectively. To demonstrate the fault-tolerant circuit, we should confirm that the probability of successful output, *F*_*p*_, in a fault-tolerant circuit (Fig. 1d) is higher than that in a non-encoded circuit (Fig. 1c), *f*_*p*_, obtained with the same hardware when correct probability p is above a threshold. The detailed calculations of *F*_*p*_ and *f*_*p*_ are presented in the supplementary information.

### Experimental setup

**Fig. 2:**
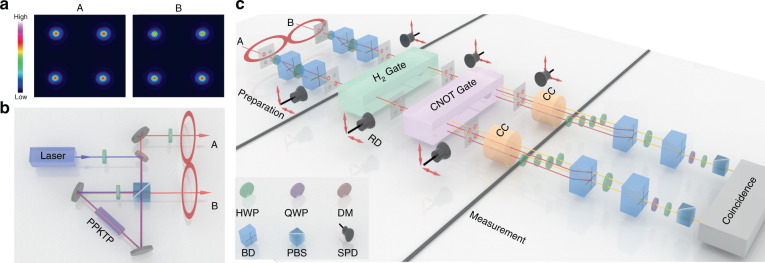
Experimental setup for verification of fault-tolerant threshold in quantum circuits. **a** Experimental images of optical spatial modes on sides of A and B is generated by exploiting a group of several beam displacers (BDs) and half-wave plates (HWPs). **b** The unit to prepare entangled photon pairs. **c** Spatial mode evolutions of fault-tolerant circuits including the stages of preparation, logical operations (H_2_ and CNOT_21_), and measurement. Output spatial modes on each side of every stage are detected with removable detectors (RDs), which are built with single-photon detectors (SPD) placed on two-dimensional movable platforms, for coincidence counts to estimate the imported error rate. Final spatial modes on each side are combined together, where optical path differences among the modes on each side are offset by compensation crystals (CC), and then measured with a quarter-wave plate (QWP), an HWP, and a polarization beam splitter (PBS). The coincidence device deals with the detected signals from two sides and outputs the coincidence count

On each side of two regions A and B, optical spatial modes, as shown in Fig. 2a, are prepared by using a group of calcite beam displacers (BDs) that separate a beam into two parallel beams with orthogonal polarizations^40,41^. Using the classical entanglement between the polarization and spatial modes of the single photon^42^ on each side, amplitudes between different spatial modes change accordingly by adjusting angles of related half-wave plates (HWPs). To generate the logical state |00〉_*l*_, spatial modes are prepared to be |00〉 and |11〉 on both sides A and B. In order to realize coincidences between |00〉_*A*_ and |00〉_*B*_, |11〉_*A*_ and |11〉_*B*_, initial entangled photons $\left| {\Phi} \right\rangle = \left( {{{{\mathrm{H}}}}_{{{\mathrm{A}}}}{{{\mathrm{H}}}}_{{{\mathrm{B}}}} + {{{\mathrm{V}}}}_{{{\mathrm{A}}}}{{{\mathrm{V}}}}_{{{\mathrm{B}}}}} \right)/\sqrt 2$, where |H〉 (|V〉) denotes horizontal (vertical) polarization of photons, are imported into A and B, respectively. With the polarization of every mode adjusted as |00〉_*A*_ = |00〉_*B*_ = |H〉 and |11〉_*A*_ = |11〉_*B*_ = |V〉, $\left| {00} \right\rangle _l \, = \left( {\left| {0000} \right\rangle + \left| {1111} \right\rangle } \right)/\sqrt 2$ is achieved, and more details are introduced in Materials and Methods.

In an experiment, as shown in Fig. 2b, a continuous-wave diode laser with a wavelength of 404 nm and a bandwidth of 0.048 nm is used to pump a 20-mm-long periodically poled KTP (PPKTP) crystal with the help of a polarized Sagnac interferometer^43^ to generate polarization-entangled photons |Φ〉. Based on this entangled source, Fig. 2c shows |00〉_*l*_ could be achieved with several BDs and HWPs which are adjusted along the preparation circuit shown in Fig. 1d. Similar implementation of logical operations H_2_ and CNOT_21_ is introduced in the  supplementary information. In the measurement stage which is constructed to measure outputs of preparation and evolution (H_2_ and CNOT_21_), four spatial modes on each side are stepwise combined together with a group of BDs and HWPs. By rotating those HWPs, related spatial modes are selected to detect with the help of a polarization analysis unit (PAU) consisting of a quarter-wave plate, an HWP, and a polarization beam splitter. At last, photons on each side are counted by a single-photon detector (SPD), and signals of two SPDs are dealt by a coincidence device. Note that during the combination process, large optical path differences occur among several beams due to the unbalanced displacement, which are compensated with birefringent crystals (see Materials and Methods).

In an experiment, the error gate is realized by adjusting the deviation of HWPs’ angles. To determine the error rate ϵ = 1 − *p* in every stage, we detect probability distributions of output spatial modes directly by a two-dimensional movable SPD. Error rate ϵ is then estimated by comparing experimental probability distributions of all modes with the ideal prediction calculated through the error model (see Materials and Methods). The probability of successful output, *F*_*p*_, can be obtained by projecting the output state onto an ideal logical state basis. Concretely, we first obtain the total coincidence count *N*_*t*_ which is the sum of eight modes with an even number of |1〉 on a physical basis, i.e., the total count in the encoded space. Output spatial modes are then projected to the ideal logical state with achieving the photon count *N*_*i*_. The successful probability *F*_*p*_ is thus given by *F*_*p*_ = *N*_*i*_/*N*_*t*_. Note that as both two counts are obtained in the same measured port, photon losses of optical elements are ignored.

**Fig. 3:**
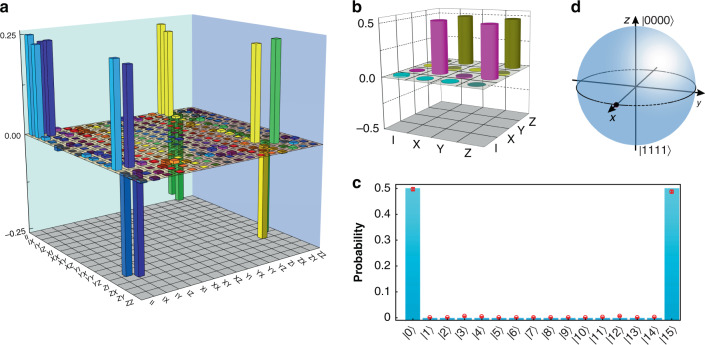
Experimental quantum process tomography of operations on physical qubits and initial logical state preparation. **a**, **b** Real parts of the experimentally reconstructed density matrix of CNOT and Hadamard operations on physical qubits, respectively, based on the Pauli operators {I, X, Y, Z}. **c** Detected probabilities of the complete basis for coincidence count of logical state |00〉_*l*_. The basis is represented with integers (e.g., |5〉 = |0101〉). Histograms and red hollow points correspond to the theoretical and experimental results. **d** Logical state $\left| {00} \right\rangle _l = \left( {\left| {0000} \right\rangle + \left| {1111} \right\rangle } \right)/\sqrt 2$. represented in the Bloch sphere on basis {|0000〉, |1111〉}. Black and pink points represent the theoretical prediction and experimental result, respectively

We first demonstrate the high performance of Hadamard and CNOT gates on physical qubits in the experiment. Quantum process tomography is performed^44,45^ (see Materials and Methods), and real parts of the experimentally reconstructed density matrix of both gates are shown in Fig. 3a, b, with fidelities of operational matrices 97.59 ± 0.01% and 99.19 ± 0.02%, respectively. The corresponding imaginary parts are small, which are illustrated in the supplementary information.

To investigate fault-tolerant circuits, we prepare a logical basis |00〉_*l*_ starting from the initial state |0000〉 along the circuit shown in Fig. 1b. Without importing error gates artificially, detected probabilities of complete basis are shown in Fig. 3c. Experimental results agree well with theoretical predictions. We deduce the inherent experimental error rate to be ϵ = 0.0007 ± 0.0001 (i.e., *p* = 0.9993 ± 0.0001). The output state, $\left| {00} \right\rangle _l \, = \left( {\left| {0000} \right\rangle + \left| {1111} \right\rangle } \right)/\sqrt 2$ which could be treated as a single qubit, is reconstructed by quantum state tomography and depicted in the Bloch sphere on a basis {|0000〉, |1111〉} with a probability of correct output 99.53 ± 0.07% (Fig. 3d). In the evolution stage consisting of operations H_2_ and CNOT_21_, the correct output probability of H_2_ reaches 99.17 ± 0.26% with success probability *p* = 0.9963 ± 0.0001. The correct output probability of CNOT_21_·H_2_ reaches 97.98 ± 0.29% with success probability *p* = 0.9945 ± 0.0001.

**Fig. 4:**
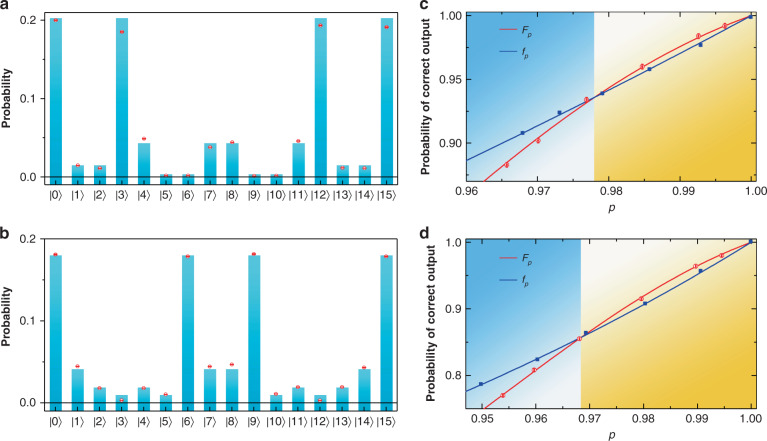
Experimental probability distributions of 16 optical modes and results of threshold. Probabilities of 16 optical modes in the outputs of the logical operation H_2_ and CNOT_21_·H_2_ are shown in **a** (*p* = 0.966) and **b** (*p* = 0.968), respectively. The histograms and red points represent the theoretical and experimental results, respectively. The x-axis represents the basis of optical modes, which are denoted in the decimal form. Panels **c**, **d** show experimental probabilities of correct output, *F*_*p*_, according to success probability *p* = 1 − ò for H_2_ and CNOT_21_·H_2_, respectively. In each panel, as shown in the right yellow region, the probability is above the threshold. Blue and red curves represent theoretical predictions of the non-encoded circuit (*f*_*p*_) and fault-tolerant circuit (*F*_*p*_), respectively. Blue squares (error bars are too small to show) and red hollow points with error bars indicate the corresponding experimental results. All error bars are estimated as standard deviations of photon counts assuming a Poisson distribution

Experimental results show that operations in this platform are extremely accurate, allowing us to observe the threshold effect in the fault-tolerant protocol. According to the introduced methods, the error rate ϵ = 1 − *p* is estimated based on experimental probability distributions of all modes. The illustrated results of ϵ = 0.034 for logical operation H_2_ and ϵ = 0.032 for CNOT_21_·H_2_ are shown in Fig. 4a, b, respectively. For the circuit implementing logical operation H_2_, the threshold is *p* = 0.978 in theory. This threshold is consistent with Fig. 4c, in which the experimental probability of correct output, *F*_*p*_, is larger than the prediction *f*_*p*_ of a non-encoded circuit detected in the same experimental platform (see more details in supplementary information) for *p* > 0.978, i.e., the right yellow region. On the other hand, when *p* < 0.978, we obtain *F*_*p*_ < *f*_*p*_. Experimental results of logical operation CNOT_21_·H_2_ are shown in Fig. 4d, in which the predicted threshold is *p* = 0.968. The experimentally obtained *F*_*p*_ is higher (lower) than the corresponding *f*_*p*_ for *p* above (below) the threshold.

It is worthy to point out that multiphoton components in prepared photon pairs would reduce the successful output probability. We also investigate the fault-tolerant threshold based on 16 spatial modes of a single photon, with detailed experimental results introduced in supplementary. Comparing results obtained by using a single photon and two entangled photons, the best probabilities of successful output states in the single-photon framework, are about *F*_*p*_ = 99.66 ± 0.07% for operation H_2_, and *F*_*p*_ = 99.33 ± 0.02% for CNOT_21_, are higher than above results. Also, error bars reduce since total counts increase in the single-photon experiment. Still and all, the fault-tolerant threshold is observed to be the same in both single-photon and two-entangled-photon experiments. Superior to the single-photon framework, the two-entangled-photon work reveals much better scalable properties of the experimental platform and provides the potential to investigate more complex fault-tolerant quantum computation.

## Discussion

Using a concise fault-tolerant protocol, we experimentally demonstrate the threshold of a complete fault-tolerant circuit with a Hadamard gate and a CNOT gate on logical qubits, besides preparing and measuring processes, with the bit-flip error in each operator. Generally, to verify a fault-tolerant protocol, the successful output probability of any circuit, formed with fault-tolerant gates, should be higher than that of the corresponding non-encoded circuit when the error rate is below a threshold. Note that rich encoding paths in the experimental setup enable different circuits for physical qubits to realize the same operations in logical space. However, only some configurations are fault-tolerant (see more analyses in supplementary information). Besides the circuit realized in this work, to implement another different circuit, we just need to rotate BDs and HWPs. And for some complicated cases, we need simply to add more sets of BDs and HWPs.

To completely demonstrate a universal fault-tolerant quantum computation remains a long-standing challenge. High-accuracy operations that can be achieved using optical systems establish an appropriate platform to simulate the error propagation in fault-tolerant circuits, especially to investigate the behavior of coherent errors^21^. Also, based on this experimental platform, nonlocal errors affecting the entangled property could be further investigated under this encoding framework. Moreover, despite the limitation of the scale of an optical system, this work facilitates the potential investigation of fault-tolerant protocols with breakthroughs of large-scale experimental implementations of quantum technology based on silicon devices^13–15^, ion-trap^46^, superconductor^47–49^, and ultra-cold atoms^50^.

Furthermore, the logical qubits constructed by physical qubits in the encoded space provide a possible method to accomplish many quantum information tasks. For example, the entanglement purification, which is able to distill the high-fidelity entangled states from the noisy low-quality entangled source^51–53^, could be achieved with the logic-qubit entanglement against bit-flip and phase-flip errors^54,55^. Another illustration is the long-distance quantum communication with logical qubits in which the fault-tolerant Calderbank–Shor–Steane encoding method is exploited to implement the ultrafast quantum communication across long distances^56–58^.

## Materials and methods

### The strategy of encoding logical qubits

According to the FT protocol in ref. ^21^, two logical qubits are encoded with four physical qubits as follows:

1 $$\begin{array}{*{20}{c}} {\left| {00} \right\rangle _l = \left( {\left| {0000} \right\rangle + \left| {1111} \right\rangle } \right)/\sqrt 2 } \\ {\left| {01} \right\rangle _l = \left( {\left| {0011} \right\rangle + \left| {1100} \right\rangle } \right)/\sqrt 2 } \\ {\left| {10} \right\rangle _l = \left( {\left| {0101} \right\rangle + \left| {1010} \right\rangle } \right)/\sqrt 2 } \\ {\left| {11} \right\rangle _l = \left( {\left| {0110} \right\rangle + \left| {1001} \right\rangle } \right)/\sqrt 2 } \end{array}$$

where {|00〉_*l*_, |01〉_*l*_, |10〉_*l*_, |11〉_*l*_} represent the logical bases, and {|0000〉, |0011〉, |0101〉, |0110〉, |1001〉, |1010〉, |1100〉, |1111〉} represent the bases of four physical qubits (the encoded space only involves even number of |1〉 in physical qubits). The four physical qubits are mapped to coincident modes of two entangled photons. As shown in Fig. 1a, with optical spatial modes on each side marked as |00〉, |01〉, |10〉, and |11〉, the basis of four physical qubits is denoted as the coincidence count between two spatial modes from the side A and B, respectively. As an illustration, the coincident mode |*mnij*〉 ≡ |*mn*〉_*A*_ ⊗ |*ij*〉_*B*_ (m, n ∈ {0, 1}_A_ and i, j ∈ {0, 1}_B_), i.e., the intensity and phase of basis |*mnij*〉 are related to the coincidence count between modes |*mn*〉_A_ and |*ij*〉_B_.

### Analysis of circuits with error gates

We introduce the detailed method of dealing with the error gates in the complete circuit. Here, taking the Hadamard operation on the second qubit in the logical space, as an illustration, the calculation is presented below. The Hadamard gate operation on the second logical qubit H_2_ is written as,

2 $${{{\mathrm{H}}}}_2 = \sigma ^I \otimes H$$

with $$\sigma ^I = \left( {\begin{array}{*{20}{c}} 1 & 0 \\ 0 & 1 \end{array}} \right)$$ being the identical operation and Hadamard gate $$H = \left( {\begin{array}{*{20}{c}} 1 & 1 \\ 1 & { - 1} \end{array}} \right)/\sqrt 2$$.

Denoting the state before H_2_ as *ρ*_hin_, and after the operation H_2_, the state becomes

3 $$\rho _{ev1} = {{{\mathrm{H}}}}_2\rho _{hin}{{{\mathrm{H}}}}_2^{\dagger}$$

Then the error gate *E*_2_ acts on the second logical qubit and the final state is

4 $$\rho _{h2} = p\rho _{ev1} + \left( {1 - p} \right)E_2\rho _{ev1}E_2^{\dagger}$$

Similar methods are employed to analyze the other error gates in the complete circuit which are introduced in the supplementary information. Also, for the fault-tolerant circuit in the physical space shown in Fig. 1b, we use the same method to calculate the corresponding result. And during the evolutions, the probability distribution of every mode can be calculated step by step.

Using the above method, we can obtain the final state after all the quantum gates including the desired gates and error gates until the stage of ideal measurement. By denoting the final experimental output state as *ρ*_out_, the success probability is achieved by projecting *ρ*_out_ to the ideal output state *ρ*_ideal_ which is the theoretical prediction in the ideal circuit without the error gates. That means the success probability is Tr[*ρ*_out_.*ρ*_ideal_].

For example, for the H_2_ operation, the success probabilities are *f*_*p*_ = *p* − 2*p*^2^ + 2*p*^3^ for the non-encoded circuit and *F*_*p*_ = *p*^2^(1 + 4*p* − 40*p*^2^ + 140*p*^3^ − 280*p*^4^ + 336*p*^5^ − 224*p*^6^ + 64*p*^7^)/(1 − 8*p* + 88*p*^2^ − 416*p*^3^ + 1104*p*^4^ − 1792*p*^5^ + 1792*p*^6^ − 1024*p*^7^ + 256*p*^8^) for the fault-tolerant encoded circuit. Comparing *f*_*p*_ and *F*_*p*_, it’s easy to find that when *p* > 0.978, *F*_*p*_ > *f*_*p*_, which means the corresponding success threshold is *p* = 0.978 and the error rate threshold is 1 − 0.978 = 0.022.

Analogously, for the CNOT_21_ *·* H_2_ operation, the success probabilities are *f*_*p*_ = *p* − 4*p*^2^ + 12*p*^3^ − 16*p*^4^ + 8*p*^5^ for the non-encoded circuit and *F*_*p*_ = *p*^2^(1 − *p* − 49*p*^2^ + 518*p*^3^ − 2884*p*^4^ + 10640*p*^5^ − 28224*p*^6^ + 55776*p*^7^ − 82880*p*^8^ + 91648*p*^9^ − 73216*p*^10^ + 39936*p*^11^ − 13312*p*^12^ + 2048*p*^13^)/(1 − 14*p* + 182*p*^2^ − 1456*p*^3^ + 8008*p*^4^ − 32032*p*^5^ + 96096*p*^6^ − 219648*p*^7^ + 384384*p*^8^ − 512512*p*^9^ + 512512*p*^10^ − 372736*p*^11^ + 186368*p*^12^ − 57344*p*^13^ + 8192*p*^14^) for the fault-tolerant encoded circuit. The success threshold is obtained as *p* = 0.968 when solving *f*_*p*_ = *F*_*p*_. And we further check that when *p* > 0.968, *F*_*p*_ > *f*_*p*_.

### The preparation of logical state |00〉_*l*_ from two entangled photons

The initial state of the optical spatial modes is in |0000〉 which equals |00〉_A_ ⊗ |00〉_B_ sharing the maximal polarization-entangled state $\left| {\Phi} \right\rangle = \left( {{{{\mathrm{H}}}}_{{{\mathrm{A}}}}{{{\mathrm{H}}}}_{{{\mathrm{B}}}} + {{{\mathrm{V}}}}_{{{\mathrm{A}}}}{{{\mathrm{V}}}}_{{{\mathrm{B}}}}} \right)/\sqrt 2$ on both sides. Following the parallel but inherently different method of the quantum walk of correlated photons^59,60^, with the help of ancillary qubit - polarization, the preparation of logical state |00〉_*l*_ starting from initial |0000〉 is introduced below.

For the initial state |0000〉 = |00〉_A_ ⊗ |00〉_B_, the polarization of the photons in the modes of |00〉_A_ and |00〉_B_ are both along horizontal (|H〉) and vertical (|V〉).

1. After the first vertical beam displacer (BD) on the side of A, the modes |00〉_A_ splits into two modes with orthogonal polarizations, i.e., |00〉_A_ in the polarization |H〉 and |10〉_A_ in the polarization |V〉. This implements a Hadamard gate on the first physical qubit leading |0000〉 to the state $(\left| {00} \right\rangle _{{{\mathrm{A}}}} \otimes \left| {00} \right\rangle _{{{\mathrm{B}}}} + \left| {10} \right\rangle _{{{\mathrm{A}}}} \otimes \left| {00} \right\rangle _{{{\mathrm{B}}}})/\sqrt 2$.
2. With a horizontal BD on A’s side, the state becomes $\left( {\left| {00} \right\rangle _{{{\mathrm{A}}}} \otimes \left| {00} \right\rangle _{{{\mathrm{B}}}} + \left| {11} \right\rangle _{{{\mathrm{A}}}} \otimes \left| {00} \right\rangle _{{{\mathrm{B}}}}} \right)/\sqrt 2$, which represents the result after the CNOT operation between the first and second physical qubits.
3. For the CNOT operation between the second and third physical qubits, a vertical BD is added on B’s side and the mode |00〉_B_ splits into two modes with orthogonal polarizations, i.e., |00〉_B_ in the polarization |H〉 and |10〉_B_ in the polarization |V〉. Due to the entangled property, the state becomes $\left( {\left| {00} \right\rangle _{{{\mathrm{A}}}} \otimes \left| {00} \right\rangle _{{{\mathrm{B}}}} + \left| {11} \right\rangle _{{{\mathrm{A}}}} \otimes \left| {10} \right\rangle _{{{\mathrm{B}}}}} \right)/\sqrt 2$.
4. Using another horizontal BD on B’ side, the final logical state |00〉_*l*_ is prepared.

And the detailed illustration including a vivid figure could be found in the supplementary information, as well as the realizations of operations H_2_ and CNOT_21_.

### Compensation of the interferometer

In the combination of optical modes, BDs are used to constitute the interferometer. For a balanced interferometer constructed by two BDs with an HWP inserted at 45°, the two beams have the same optical lengths. Since these two beams are close to each other and suffer the same environmental noise, this kind of interferometer is inherently stable. While for an unbalanced interferometer, a compensation crystal (CC) is placed on the path with a shorter optical length. In an experiment, the optical path difference between two beams from a BD with a length of 28.3 mm is about 2.28 mm. A length of 4-mm Lithium niobate (LiNbO3) crystal and several quartz plates are exploited as the CC inserted in the deflected beam to compensate for the different optical lengths. In our work, the visibility of the interferometer is very high to ensure that experimental measurement is implemented with a successful probability of 99.2–99.8% compared with the ideal project measurement. Note that, the part caused by the error rate is not compensated in the measurement and as a result, the decoherence between this error part and other successful modes will exist to match the error mode introduced in section I of supplementary information.

### Estimation of the value ϵ

In the experiment, the error rate ϵ is imported by adjusting the deviation of HWPs’ angle. All intensities of the 16 coincident modes are detected with the two-dimensional movable detector on each side, which is shown in Figs. 3c, 4a, b, to obtain the corresponding probability distributions. The experimental probability distributions of all coincident modes are used to estimate the error rate $\epsilon$ by comparing with the ideal prediction calculated through the error model introduced in section I of supplementary information.

### Details to perform the quantum process tomography

For the detailed tomography for a quantum process ε with denoting the process density matrices χ, the output of this process could be represented as ε(ρ) = ∑_m,n_χ_mn_E_m_ρE_n_^†^ for the input state ρ, where E_m_ is one of the Pauli operators {I, X, Y, Z}. For the n-qubit density matrices, the number of elements χ_mn_ is 2^2n^. Scanning the input state ρ and performing the state tomography on the corresponding output state *ε*(*ρ*), we can reconstruct the density matrices χ with the maximum-likelihood method. In detail, for a single-qubit quantum gate, first, we prepare the six eigenstates of Pauli vectors, i.e., the states |H〉, |V〉, $\left( {\left| {{{\mathrm{H}}}} \right\rangle \pm \left| {{{\mathrm{V}}}} \right\rangle } \right)/\sqrt 2$, $\left( {\left| {{{\mathrm{H}}}} \right\rangle \pm i\left| {{{\mathrm{V}}}} \right\rangle } \right)/\sqrt 2$, as the input states. Then, for every input state, we perform the state tomographic measurement on the output state. At last, with the complete information of input and output states, we reconstruct the density matrices χ of the quantum gate based on the Pauli operators {I, X, Y, Z} using the maximum-likelihood technique. For a two-qubit quantum gate, the input states are set as the product states |A〉|B〉, where |A〉 and |B〉 are the six Pauli eigenstates on the regions of A and B, respectively. And the other steps are similar to the above description. Comparing the experimentally reconstructed density matrices χ_exp_ with the theoretical prediction χ_theo_, we can achieve the fidelity *Fide* = Tr[χ_exp_.χ_theo_].

## Supplementary information

Supplementary Information for Optical demonstration of quantum fault-tolerant threshold

## Data Availability

Source data are available for this paper. All other data that support the plots within this paper and other findings of this study are available from the corresponding author on reasonable request.
