# Supplementary material for: Optical demonstration of quantum fault-tolerant threshold
Source: Light Sci Appl. 2022 Jul 5;11:203. doi: 10.1038/s41377-022-00891-9 (PMC9256730; doi:10.1038/s41377-022-00891-9)
Supplement: Supplementary file 1 — Supplementary Information for Optical demonstration of quantum fault-tolerant threshold [file 41377_2022_891_MOESM1_ESM.pdf]

Supplementary Information for
Optical demonstration of quantum fault-tolerant threshold

Kai Sun,[1,2,3] Ze-Yan Hao,[1,2,3] Yan Wang,[1,2,3] Jia-Kun Li,[1,2,3] Xiao-Ye Xu,[1,2,3] Jin-Shi Xu,[1,2,3,*] Yong-Jian Han,[1,2,3,†] Chuan-Feng Li,[1,2,3,‡] and Guang-Can Guo[1,2,3]

[1]*CAS Key Laboratory of Quantum Information, University of Science and Technology of China, Hefei 230026, China*

[2]*CAS Center for Excellence in Quantum Information and Quantum Physics, University of Science and Technology of China, Hefei 230026, China*

[3]*Hefei National Laboratory, University of Science and Technology of China, Hefei 230088, China*

(Dated: June 9, 2022)

* jsxu@ustc.edu.cn

† smhan@ustc.edu.cn

‡ cfli@ustc.edu.cn

CONTENTS

I. Theoretical analysis of fault-tolerant circuits 2
A. The non-encoded protocol for the logical operation H_2 with error gates 2
B. Fault-tolerant circuits with error gates 3
C. Analysis of different circuits 4
D. Different error gates 6
E. Different input state 6

II. More experimental details 7
A. The preparation of logical state $|00\rangle_l$ from two entangled photons 7
B. The realization of operation H_2 8
C. The realization of operation CNOT_{21} 9
D. Compensation of the interferometer 10
E. More results of quantum process tomography 11
F. Method to detect the probability of f_p in the non-encoded circuits 12

III. Experimental results in the single-photon framework 13

I. THEORETICAL ANALYSIS OF FAULT-TOLERANT CIRCUITS

A. The non-encoded protocol for the logical operation H_2 with error gates

First, we consider the non-encoded protocol in which the Hadamard operation is performed on a two-qubit state. The circuit is shown in Fig. 1**c** in the main text. The initial state could be written $\rho_{in} = |00\rangle\langle 00|$. The Hadamard gate operation on the second qubit H_2 is written as

$$\mathrm{H}_2 = \sigma^I \otimes H, \tag{1}$$

with $\sigma^I = \left(\begin{smallmatrix}1 & 0\\ 0 & 1\end{smallmatrix}\right)$ being the identical operation and Hadamard gate $H = \left(\begin{smallmatrix}1 & 1\\ 1 & -1\end{smallmatrix}\right)/\sqrt{2}$. For the ideal situation without error gates, the final state is $\rho_{ideal} = \mathrm{H}_2 \cdot \rho_{in} \cdot \mathrm{H}_2^{\dagger}$.

Considering the error gate $E = \sigma^x$ illustrated in Fig. 1**c** occurring in all the processes of preparation, evolution and measurement, the subsequent evolved state before operation H_2

is

$$\rho_{in1} = p\rho_{in} + (1-p)E_1\rho_{in}E_1^\dagger,$$
$$\rho_{in2} = p\rho_{in1} + (1-p)E_2\rho_{in1}E_2^\dagger, \quad (2)$$

where $1-p=\epsilon$ is the error rate of the error gate E and p represets the success probability. $E_1 = \sigma^x \otimes \sigma^I$ and $E_2 = \sigma^I \otimes \sigma^x$ are the error gates implemented on the first and second qubits, respectively.

For the operation of H_2, the error gate acts on the second qubit. The evolution of state becomes

$$\rho_{ev1} = \mathrm{H}_2\rho_{in2}\mathrm{H}_2^\dagger,$$
$$\rho_{ev2} = p\rho_{ev1} + (1-p)E_2\rho_{ev1}E_2^\dagger. \quad (3)$$

In the measurement process, both qubits are affected by the error gates. And the output state ρ_{out} before the ideal measurement is

$$\rho_{m1} = p\rho_{ev2} + (1-p)E_1\rho_{ev2}E_1^\dagger,$$
$$\rho_{m2} = p\rho_{m1} + (1-p)E_2\rho_{m1}E_2^\dagger, \quad (4)$$
$$\rho_{out} = \rho_{m2}.$$

By projecting output state ρ_{out} on the ideal output state ρ_{ideal}, the correct probability $f_p = \text{Tr}[\rho_{\text{out}}.\rho_{\text{ideal}}]$ is theoretically calculated to $f_p = p - 2p^2 + 2p^3$.

B. Fault-tolerant circuits with error gates

For the fault-tolerant (FT) circuit shown in Fig. 1**d** in the main text. The correct probability F_p is obtained in a similar way of the above non-encoded circuits by projecting the output state ρ_{out} on the ideal measurement state ρ_{ideal}.

For the preparation of logical state $|00\rangle_l$ whose circuit is shown in Fig. 1**b** in the main text, following the error gates and measurement shown in Fig. 1**d**, we can calculate the corresponding threshold about $p = 0.986$. The results of $D_p = F_p - f_p$ are shown in Fig. S1.

For the operation of Hadamard gate on the second logical qubit, compared with the theoretical prediction of non-encoded protocol, F_p is larger than f_p for $p > 0.978$. The threshold error rate ϵ is about 0.022. The comparison of f_p and F_p is shown in Fig. 4**a** in the main text.

We further investigate the operation of $\mathrm{CNOT}_{21}\cdot\mathrm{H}_2$ in which $f_p = p-4p^2+12p^3-16p^4+8p^5$ for the non-encoded circuit. The comparison of f_p and F_p is shown in Fig. 4**b** with the threshold being 0.968.

During the evolution, the probability distribution of every mode can be calculated step by step.

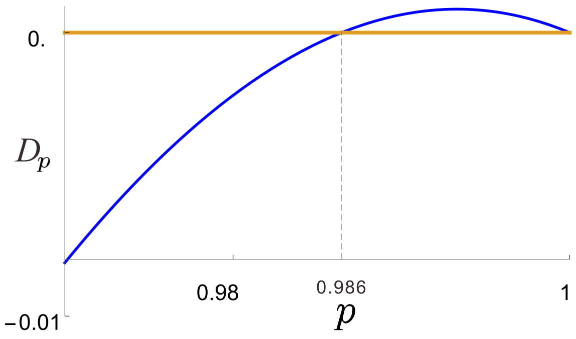

FIG. S1. The difference $D_p = F_p - f_p$ between the correct probabilities of the fault-tolerant circuit and the non-encoded circuit for the preparation of logical state $|00\rangle_l$. The threshold appears at p=0.986.

C. Analysis of different circuits

Based on the preparation circuit introduced in the main text, other three circuits to implement H_2 are shown in Fig. S2**a-c**. In these circuits, the operations on the four physical qubits are different from that shown in main text. By following the similar method introduced above to implement the error gate $E = \sigma^x$, theoretical predictions F_p could be obtained. The corresponding differences between the correct probability of FT and non-encoded circuits $D_p = F_p - f_p$ are shown under Fig. S2**a-c**. For these circuits with error gates, different output states are generated and the corresponding thresholds sensibly change. For the circuits in Fig. S2**b** and **c**, D_p is always less than 0 ($D_p = 0$ for the trival cases with $p = 0$ and 1), which means that there is no advantage for employing such encoded circuits.

We further consider different circuits to implement the logical operation $\mathrm{CNOT}_{21}\cdot\mathrm{H}_2$ which is shown in Fig. S3. D_p is always smaller than zero except for the trivial cases with $p = 0$ and 1. As a result, there is no advantage for such encoded circuit.

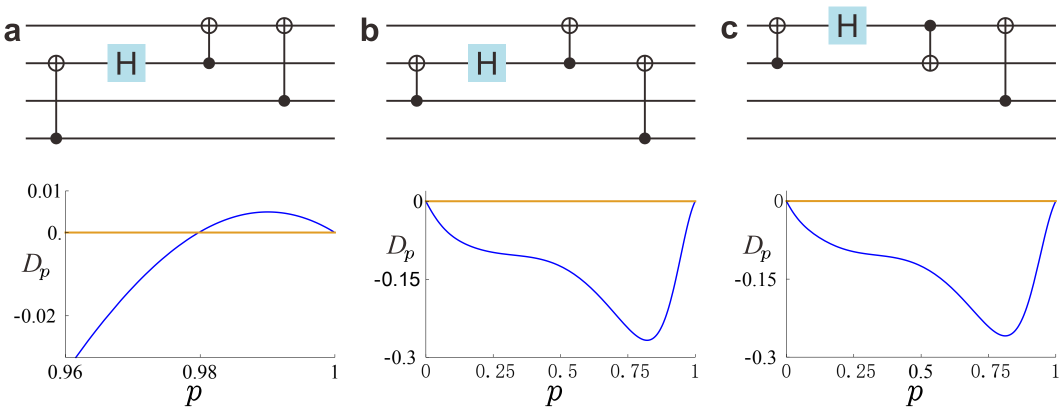

FIG. S2. Different circuits to implement the logical operation H_2. The difference $D_p = F_p - f_p$ between the correct probabilities of the fault-tolerant circuit and the non-encoded circuit is shown below the corresponding circuit. The blue lines represent the theoretical results and the yellow lines represent the boundary of zero. The threshold in **a** appears at p=0.98. While there are no thresholds in **b** and **c**.

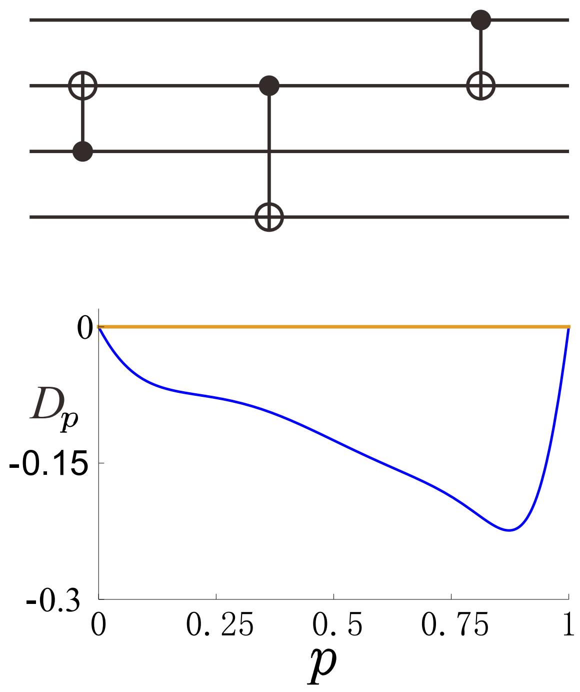

FIG. S3. The circuit to implement logical operation CNOT_{21}. The difference $D_p = F_p - f_p$ is shown below the corresponding circuit. The blue line represent the theoretical result and the yellow line represent the boundary of zero.

D. Different error gates

Under the framework of encoding rules in the main text, we can investigate the gates, for both operation gates and error gates, which contribute the flip of qubit. For the phase gate which affects the phase between different kets, the number of 1s of the basis would not change, which leads all the physical states inside the encoding space. To clarify this point, we further consider two other types of error gate σ^y and σ^z to investigate their effect on the threshold of the FT protocol. The used encoded circuit is same as that used in the main text for the Hadamard gate H_2. Fig. S4**a** shows the theoretical results of $D_p = F_p - f_p$ with a threshold of $p = 0.983$ for the error gate $E = \sigma^y$. Fig. S4**b** shows the corresponding results when the error gate $E = \sigma^z$. Since the difference D_p is smaller than zero for $p \in (0, 1)$, there is no advantage for the encoded circuit compared with non-encoding circuit with σ^z errors, which means the circuit is not FT.

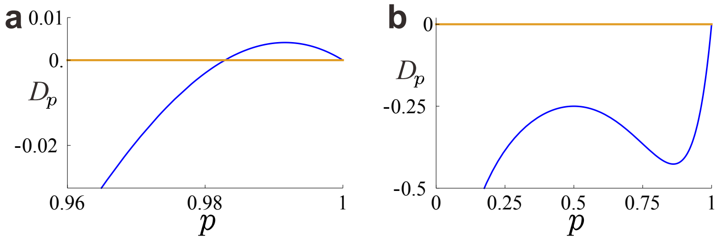

FIG. S4. **a**, **b** show the theoretical results of $D_p = F_p - f_p$ for $E = \sigma^y$ and $E = \sigma^z$, respectively. The threshold in **a** is about $p = 0.983$.

E. Different input state

In our work, the input physical state starts from the generally initial one $|0000\rangle$. As mentioned in the main text, in our special encoding, only Clifford gates, such as CNOT, Hadamard and X gates, can be implemented as the FT manner. With these FT gates, the logical input can be prepared as state $|00\rangle_l$. Obviously, we can implement the FT gates on the initial state $|00\rangle_l$ and transform it to some new input states. For example, to get the input state $|10\rangle_l$, we just perform X_1 gate on the first logical qubit. We need to note that for each circuit formed with the FT gates, there may exist a different error rate threshold. As a result, compared with the circuits, $\mathrm{CNOT}_{21} \cdot \mathrm{H}_2$ in our experiment, for the input state

$|10\rangle_l$, the threshold will change since the X_1 gate could be treated as another logical circuit, which means the whole logical circuits $\mathrm{CNOT}_{21} \cdot \mathrm{H}_2 \cdot X_1$.

II. MORE EXPERIMENTAL DETAILS

A. The preparation of logical state $|00\rangle_l$ from two entangled photons

Two polarized-entangled photons are sent to two sides, A and B, as shown in the Fig. **1a** in the main text. The optical spatial modes are marked as $|00\rangle, |01\rangle, |10\rangle, |11\rangle$ on each side. The basis of four physical qubits is denoted as the combination of spatial modes on the sides of A and B. Here, $|mnij\rangle \equiv |mn\rangle_\mathrm{A} \otimes |ij\rangle_\mathrm{B}$ ($m, n \in \{0,1\}_\mathrm{A}$ and $i, j \in \{0,1\}_\mathrm{B}$).

The initial state of the optical spatial modes is in $|0000\rangle$ which equals $|00\rangle_\mathrm{A} \otimes |00\rangle_\mathrm{B}$ sharing the maximally polarization entangled state $|\Phi\rangle = (|\mathrm{H_A H_B}\rangle + |\mathrm{V_A V_B}\rangle)/\sqrt{2}$ on both sides. With the help of ancillary qubit - polarization, the preparation of logical state $|00\rangle_l$ starting from initial $|0000\rangle$ is illustrated in Fig. S5.

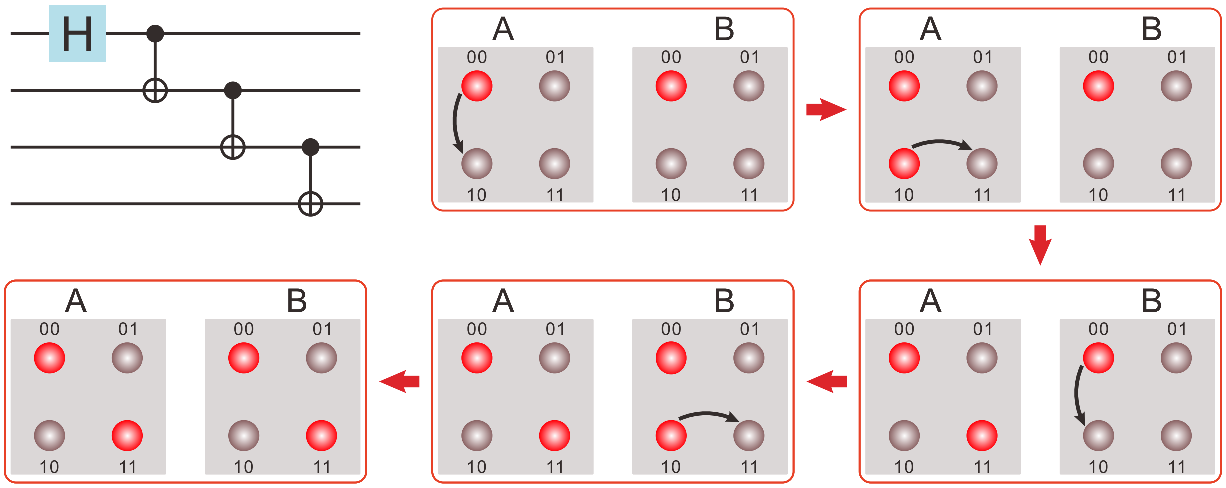

FIG. S5. Evolution of the spatial modes on both sides in the preparation of logical state $|00\rangle_l$ starting from initial $|0000\rangle$.

For the initial state $|0000\rangle = |00\rangle_\mathrm{A} \otimes |00\rangle_\mathrm{B}$, the polarization of the photons in the modes of $|00\rangle_\mathrm{A}$ and $|00\rangle_\mathrm{B}$ are both along horizontal ($|\mathrm{H}\rangle$) and vertical ($|\mathrm{V}\rangle$).

1. After the first vertical beam displacer (BD) on the side of A, the modes $|00\rangle_\mathrm{A}$ splits into two modes with orthogonal polarizations, i.e., $|00\rangle_\mathrm{A}$ in the polarization $|\mathrm{H}\rangle$ and

$|10\rangle_A$ in the polarization $|V\rangle$. This implements a Hadamard gate on the first physical qubit leading $|0000\rangle$ to the state $(|00\rangle_A \otimes |00\rangle_B + |10\rangle_A \otimes |00\rangle_B)/\sqrt{2}$.

2. With a horizontal BD in A's side, the state becomes $(|00\rangle_A \otimes |00\rangle_B + |11\rangle_A \otimes |00\rangle_B)\sqrt{2}$, which represents the result after the CNOT operation between the first and second physical qubits.

3. For the CNOT operation between the second and third physical qubits, a vertical BD is added on B's side and the mode $|00\rangle_B$ splits into two modes with orthogonal polarizations, i.e., $|00\rangle_B$ in the polarization $|H\rangle$ and $|10\rangle_B$ in the polarization $|V\rangle$. Due to the entangled property, the state becomes $(|00\rangle_A \otimes |00\rangle_B + |11\rangle_A \otimes |10\rangle_B)/\sqrt{2}$.

4. Using another horizontal BD on B' side, the final logical state $|00\rangle_l$ is prepared.

B. The realization of operation H_2

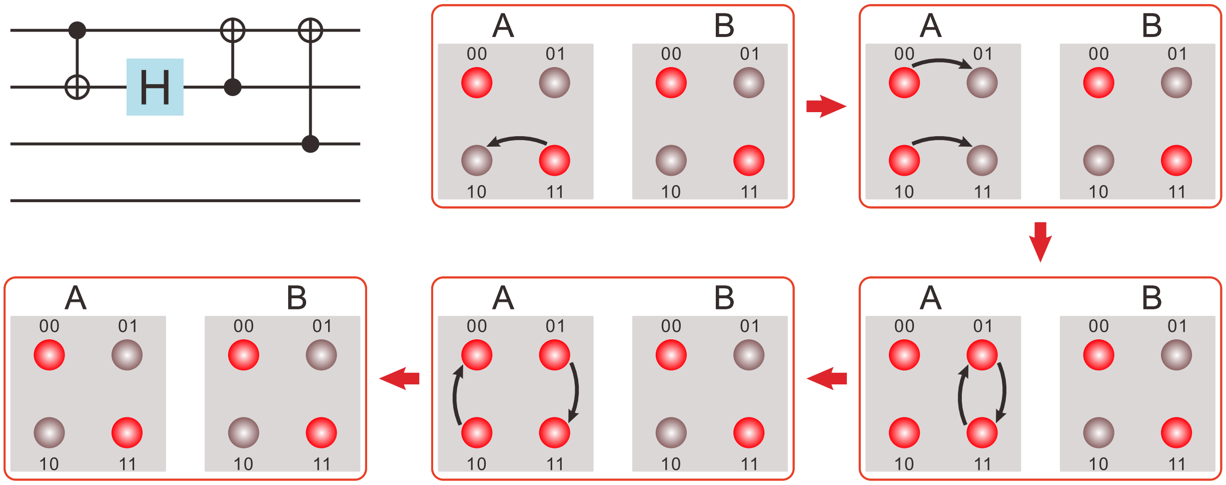

FIG. S6. Evolution of the spatial modes on both sides for the logical operation H_2 leading the logical state $|00\rangle_l$ to be $(|00\rangle_l + |01\rangle_l)/\sqrt{2}$.

Here, we introduce the evolutions of spatial modes in the realization of logical operation H_2. As shown in Fig. S6, based on the prepared state $|00\rangle_l = (|00\rangle_A \otimes |00\rangle_B + |11\rangle_A \otimes |11\rangle_B)/\sqrt{2}$, the evolutions of spatial modes are introduced below.

1. After the physical operation $CNOT_{12}$, the coincident modes become as $(|00\rangle_A \otimes |00\rangle_B + |10\rangle_A \otimes |11\rangle_B)/\sqrt{2}$.

2. It is adjusted as $(|00\rangle_A \otimes |00\rangle_B + |01\rangle_A \otimes |00\rangle_B + |10\rangle_A \otimes |11\rangle_B + |11\rangle_A \otimes |11\rangle_B)/2$ after the Hadamard operation on the second physical qubit.

3. The following physical CNOT_{21} operation leads the modes $|01\rangle_A$ and $|11\rangle_A$ exchange.

4. And the last operation CNOT_{31} leads the modes $|01\rangle_A$ to $|11\rangle_A$, and $|10\rangle_A$ to $|00\rangle_A$.

As a result, the final modes on side A are $|00\rangle_A$ and $|11\rangle_A$, both of which own the polarizations $|H\rangle$ and $|V\rangle$, and the modes on side B remain unchanged, i.e., $|00\rangle_B$ owns the photon of $|H\rangle$ and $|11\rangle_A$ owns the photon of $|V\rangle$. Thus, the coincident modes are $(|00\rangle_A \otimes |00\rangle_B + |00\rangle_A \otimes |11\rangle_B + |11\rangle_A \otimes |00\rangle_B + |11\rangle_A \otimes |11\rangle_B)/2$ which is the logical state $(|00\rangle_l + |01\rangle_l)/\sqrt{2}$.

C. The realization of operation CNOT_{21}

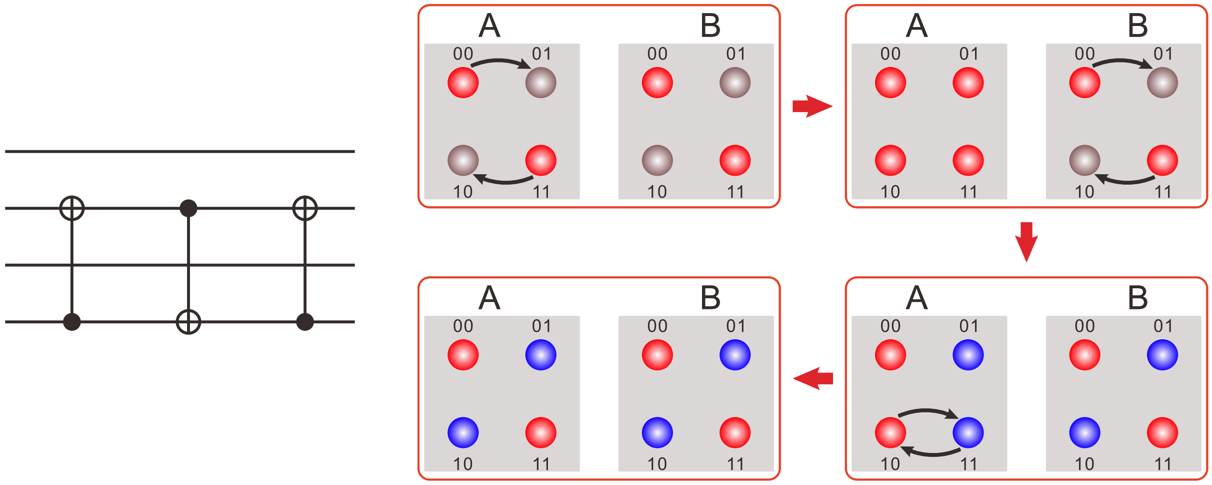

FIG. S7. Evolution of the spatial modes on both sides for the logical operation CNOT_{21} leading the logical state $(|00\rangle_l + |01\rangle_l)/\sqrt{2}$ to be $(|00\rangle_l + |11\rangle_l)/\sqrt{2}$. Only the same color modes on two sides have the coincidence count.

Fig. S7 introduces the circuit of implementing the logical operation CNOT_{21} and the corresponding evolution of both sides' spatial modes. Here, the detailed process is explained below.

1. From the output state of logical operation H_2, the spatial modes on side A are changed to be $|00\rangle_A$ with polarization $|H\rangle$, $|01\rangle_A$ with polarization $|V\rangle$, $|10\rangle_A$ with polarization

$|\mathrm{V}\rangle$, and $|11\rangle_\mathrm{A}$ with polarization $|\mathrm{H}\rangle$ after the first physical operation CNOT. According to the entangled property $(|\mathrm{H_AH_B}\rangle+|\mathrm{V_AV_B}\rangle)/\sqrt{2}$, this will lead that the coincident modes are $(|00\rangle_\mathrm{A}\otimes|00\rangle_\mathrm{B}+|01\rangle_\mathrm{A}\otimes|11\rangle_\mathrm{B}+|11\rangle_\mathrm{A}\otimes|00\rangle_\mathrm{B}+|10\rangle_\mathrm{A}\otimes|11\rangle_\mathrm{B})/2$.

2. After the second physical CNOT operation, the coincident modes are predicted as $(|00\rangle_\mathrm{A}\otimes|00\rangle_\mathrm{B}+|01\rangle_\mathrm{A}\otimes|10\rangle_\mathrm{B}+|11\rangle_\mathrm{A}\otimes|01\rangle_\mathrm{B}+|10\rangle_\mathrm{A}\otimes|11\rangle_\mathrm{B})/2$. To realize this aim, the modes $|01\rangle_\mathrm{B}$ (polarization $|\mathrm{H}\rangle$) comes from $|00\rangle_\mathrm{B}$, and $|10\rangle_\mathrm{B}$ (polarization $|\mathrm{V}\rangle$) comes from $|11\rangle_\mathrm{B}$. Moreover, to obtain the desired coincident modes, the modes $|01\rangle_\mathrm{A}$ and $|11\rangle_\mathrm{A}$ on side A are delayed a time t, which is longer than the coincident window, to the other modes on this side, and the modes $|01\rangle_\mathrm{B}$ and $|10\rangle_\mathrm{B}$ on side B are also delayed the same time. The delayed modes are marked with blue color to distinguish the other four modes. As a result, the coincident modes are $|00\rangle_\mathrm{A}\otimes|00\rangle_\mathrm{B}$ ($|H_AH_B\rangle$), $|10\rangle_\mathrm{A}\otimes|11\rangle_\mathrm{B}$ ($|\mathrm{V_AV_B}\rangle$), $|01\rangle_\mathrm{A}\otimes|10\rangle_\mathrm{B}$ (delayed $|\mathrm{V_AV_B}\rangle$), and $|11\rangle_\mathrm{A}\otimes|01\rangle_\mathrm{B}$ (delayed $|\mathrm{H_AH_B}\rangle$).

3. The three physical CNOT operation leads the modes $|10\rangle_\mathrm{A}$ and $|11\rangle_\mathrm{A}$ exchange with each other. At last, the coincident modes are $|00\rangle_\mathrm{A}\otimes|00\rangle_\mathrm{B}$ ($|\mathrm{H_AH_B}\rangle$), $|11\rangle_\mathrm{A}\otimes|11\rangle_\mathrm{B}$ ($|\mathrm{V_AV_B}\rangle$), $|01\rangle_\mathrm{A}\otimes|10\rangle_\mathrm{B}$ (delayed $|\mathrm{V_AV_B}\rangle$), and $|10\rangle_\mathrm{A}\otimes|01\rangle_\mathrm{B}$ (delayed $|\mathrm{H_AH_B}\rangle$).

In theory, the delayed time will be compensated before the measurement stage and the state after this logical operation CNOT_{21} could be written as $(|00\rangle_\mathrm{A}\otimes|00\rangle_\mathrm{B}+|11\rangle_\mathrm{A}\otimes|11\rangle_\mathrm{B}+|01\rangle_\mathrm{A}\otimes|10\rangle_\mathrm{B}+|10\rangle_\mathrm{A}\otimes|01\rangle_\mathrm{B})/2=(|00\rangle_l+|11\rangle_l)/\sqrt{2}$. Therefore, we can set the delayed time in the experimental detection of probability distribution of coincident modes, with delaying the signals of related spatial modes (blue color modes). In experiment, the coincident window is set as 1 ns and the delayed time $t=3$ ns.

D. Compensation of the interferometer

In the combination of optical modes, beam displacers (BDs) are used to constitute the interferometer. As shown in Fig. S8, in a balanced interferometer (shown in Fig. S8**a**) constructed by two BDs with a half wave plate (HWP) inserted at 45°, the two beams have the same optical lengths. Since these two beams are close to each other which suffers the same environmental noise, this kind of interferometer is inherently stable. While for the unbalanced interferometer shown in Fig. S8**b**, a compensation crystal (CC) is placed on the

path with a shorter optical length. In experiment, the optical path difference between two beams from a BD with a length of 28.3 mm is about 2.28 mm. A length of 4 mm Lithium niobate ($LiNbO_3$) crystal and several quartz plates are exploited as the CC inserted in the deflected beam to compensate the different optical length. In our work, the visibility of interferometer is very high to ensure that experimental measurement is implemented with a successful probability of 99.2-99.8% compared with the ideal project measurement. Note that, the part caused by the error rate is not compensated in the measurement and as a result, the decoherence between this error part and other successful modes will exist to match the error mode introduced in the above section I.

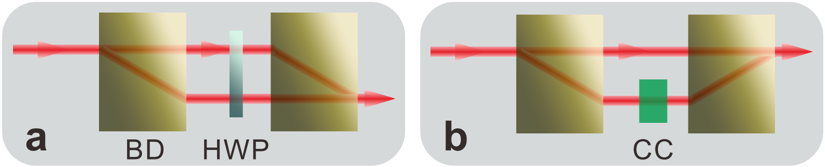

FIG. S8. **a**. Illustration of a balanced interferometer built up by two beam displacers (BDs) with a half wave plate (HWP) set at 45°. **b**. An unbalanced interferometer with compensation crystal (CC) inserted.

E. More results of quantum process tomography

The imaginary parts of density matrixes of Hadamard gate and CNOT gate on physical qubits based on the operational basis $\{I,\ X,\ Y,\ Z\}$ are shown in Fig. S9. For the gate fidelities measured by quantum process tomography, the results are obtained by comparing the experimental-reconstructed density matrices of gates with the ideal matrices. It is quite different from the method of estimation of ϵ. In the estimation of ϵ, as mentioned above, the probability distributions of every mode, especially the probabilities of the modes owning odd number of $|1\rangle$, play the core role. Since some modes will be projected outside the code-space, the coherent interactions between different modes are absent in the estimation of ϵ. While for the gate fidelities, the coherence of different modes plays the key role. To obtain the predicted outputs, the measurements are more complex and are similar with the projective measurement on the logical state shown in Fig. 2**c** in the main text. We need to compensate the optical path differences among the modes to constitute the interferometers

which fulfill the corresponding coherence. And the imperfect interferometers in experiment affect greatly the quantum process tomography, which leads to lower the fidelities of gates.

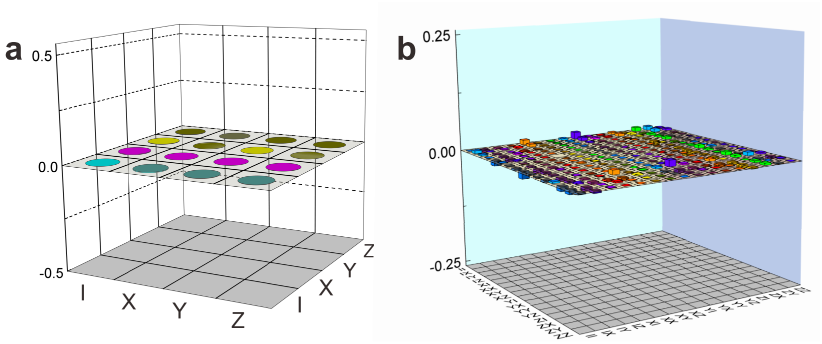

FIG. S9. The experimental imaginary parts of density matrixes of Hadamard gate (**a**) and CNOT gate (**b**) on physical qubits based on the operational basis $\{I,\ X,\ Y,\ Z\}$.

F. Method to detect the probability of f_p in the non-encoded circuits

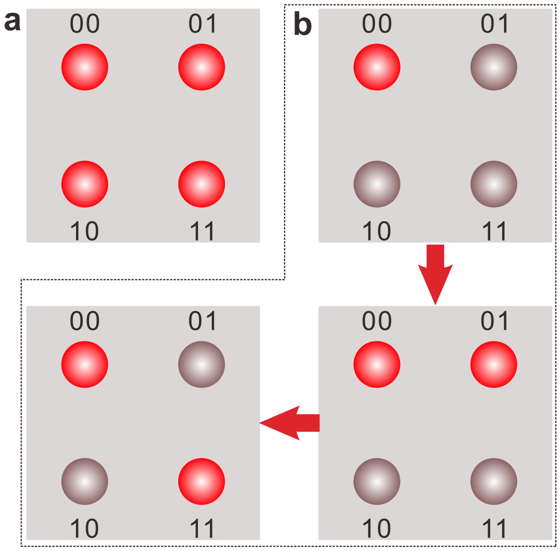

FIG. S10. **a** The mapping of logical qubits to the spatial modes of a single photon. **b** The detailed evolutions of modes for the operation Hadamard gate H_2 and CNOT gate $CNOT_{21}$.

In the same experimental platform, we measure the probability of correct output f_p in the non-encoded circuits. As shown in Fig. S10**a**, the two logical qubits are mapped directly to the four spatial modes of a single photon. Following the operations H_2 and $CNOT_{21}$,

the evolutions of modes are illustrated in Fig. S10**b**. With the measurement onto the ideal projector $(|00\rangle_l + |01\rangle_l)/\sqrt{2}$ and $(|00\rangle_l + |11\rangle_l)/\sqrt{2}$ (see the similar detailed description in Section III below), respectively, the value of f_p is achieved for a certain error rate $\epsilon = 1 - p$.

III. EXPERIMENTAL RESULTS IN THE SINGLE-PHOTON FRAMEWORK

We further investigate the FT threshold based on 16 spatial modes of a single photon in the similar experimental setup, as shown in Fig. S11. Different from the two-entangled-photon experiment which only uses a type of beam displacer (BD), BD30 separating two beams with the distances 3.0 mm, here, another type of beam displacer (BD) are used at the same time, which is BD60 separating two beams with the distances 6.0 mm. In experiment, attenuated laser pulses, whose width is about 130 fs and repetition rate is about 76 MHz at the wavelength of 800 nm, with about 0.004 photons averagely in every pulse are used as the photon source of input beam.

Following the similar framework in the main text, using the classical entanglement between the polarizations and spatial modes of the single photon, amplitudes between different spatial modes change accordingly by adjusting angles of related half-wave plates (HWPs). The detailed process could be described as following. When the spatial mode $|m\rangle$ with the polarization $|\mathrm{H}\rangle$ is transferred to the superposed mode $(|m\rangle + |n\rangle)/\sqrt{2}$, we first rotate the polarization from $|\mathrm{H}\rangle$ to $(|\mathrm{H}\rangle + |\mathrm{V}\rangle)/\sqrt{2}$ with an HWP, leading to the system's state as $|m\rangle \otimes (|\mathrm{H}\rangle + |\mathrm{V}\rangle)/\sqrt{2}$. Then passing a beam displacer which separates a spatial mode into two parallel modes with orthogonal polarizations, we obtain the state $(|m\rangle \otimes |\mathrm{H}\rangle + |n\rangle \otimes |\mathrm{V}\rangle)/\sqrt{2}$, which is the classical entanglement between the polarizations and spatial modes of a single photon. At last, we need to reverse the polarization of the photon located at the spatial mode $|n\rangle$ and achieve the desired superposed mode.

The experimental intensity image is shown in Fig. S11**a**. Following the same encoded FT circuits in the main text, the preparation and logical operations (H_2 and CNOT_{21}) are realized with corresponding changes of the 16 spatial modes.

Note that in experiment, we project the final state to the logical basis by combining corresponding spatial modes and projecting to a proper polarization state. For example, by setting the spatial modes $|0000\rangle$ and $|1111\rangle$ to be horizontal ($|\mathrm{H}\rangle$) and vertical ($|\mathrm{V}\rangle$) polarizations, respectively, the correct probability of $|00\rangle_l = (|0000\rangle + |1111\rangle)/\sqrt{2}$ can be

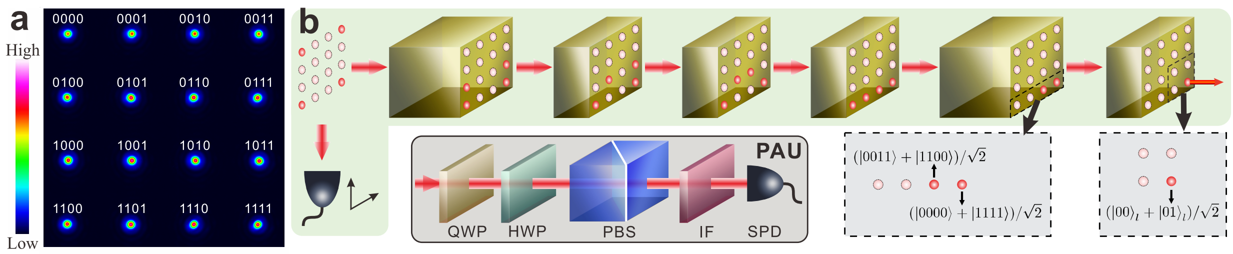

FIG. S11. Experimental setup for verification of fault-tolerant threshold in quantum circuits with a single photon. **a**. Experimental image of optical spatial modes generated by exploiting a group of several BDs with different lengths (BD30 and BD60) and HWPs. **b**. Evolution of spatial modes for measurement at output state $(|00\rangle_l + |01\rangle_l)/\sqrt{2}$ after applying H_2. A single-photon detector (SPD) equipped with an interference filter (IF) (not shown here) is placed on a two-dimensional movable platform to scan and detect the intensity of the 16 spatial modes. The explicit expression of the spatial modes at the last two steps are shown in the dashed panels. The coherent information in the spatial modes is reflected in the polarization information, which is analyzed by a polarization analysis unit (PAU) consisting of a quarter-wave plate (QWP), a HWP, and a polarization beam splitter (PBS).

obtained by combining the two modes together and projecting on the polarization state $(|\mathrm{H}\rangle + |\mathrm{V}\rangle)/\sqrt{2}$ using the polarization analysis unit. For $(|00\rangle_l + |01\rangle_l)/\sqrt{2}$, we need to combine the modes $|0000\rangle$ and $|1111\rangle$ (the logical state $|00\rangle_l$), and the modes $|0011\rangle$ and $|1100\rangle$ (the logical state $|01\rangle_l$), respectively. If the phase information between $|0000\rangle$ and $|1111\rangle$ keeps stable, the state $|00\rangle_l$ which is originally in the polarization $(|\mathrm{H}\rangle + |\mathrm{V}\rangle)/\sqrt{2}$ could be set to be $|\mathrm{H}\rangle$, while $|01\rangle_l$ could be reset to be $|\mathrm{V}\rangle$. Combining both two new modes to be $(|\mathrm{H}\rangle + |\mathrm{V}\rangle)/\sqrt{2}$, we then project the state to $(|00\rangle_l + |01\rangle_l)/\sqrt{2}$.

We then implement fault-tolerant circuits for different quantum gate operations. Without implementing error gates, the probability of correct output of Hadamard operation H_2 reaches $99.66 \pm 0.07\%$ with success probability $p = 0.9988 \pm 0.0001$ (i.e., error rate $\epsilon = 0.0012 \pm 0.0001$). The probability of correct output of $\mathrm{CNOT}_{21} \cdot H_2$ reaches $99.33 \pm 0.02\%$ with success probability $p = 0.9997 \pm 0.0001$ (i.e., error rate $\epsilon = 0.0003 \pm 0.0001$). Importing the extremely accurate error rate ϵ, we could observe the threshold effect in the fault-tolerant protocol and the experimental results are shown in Fig. S12 including the comparison with the results obtained in the two-entangled-photon experiment. It's obvious to achieve the

conclusion that the results in single-photon and two-entangled-photon frameworks are the same for the FT circuits.

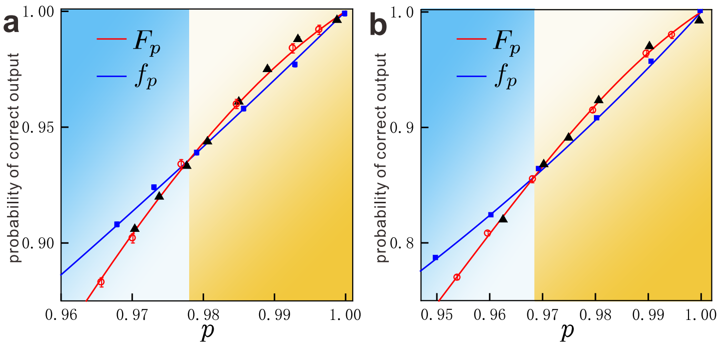

FIG. S12. Experimental probabilities of correct output for different operations. Panels **a** and **b** show experimental probabilities of correct output, F_p, according to success probability $p = 1 - \epsilon$ for H_2 and $CNOT_{21} \cdot H_2$, respectively. The blue and red curves represent the theoretical predictions of the non-encoded circuit (f_p) and fault-tolerant circuit (F_p). The black triangles and red hollow points indicate the experimental results achieved in the single-photon and two-photon experiments, respectively. While the blue squares are the experimental results for the non-encoded circuits. The errorbars of blue squares and black triangles are too small to see. All error bars are estimated as standard deviations of the photon counts assuming a Poisson distribution.
